# Unconventional Anomalous Hall Effect Driven by Self‐Intercalation in Covalent 2D Magnet Cr_2_Te_3_

**DOI:** 10.1002/advs.202407625

**Published:** 2024-11-25

**Authors:** Keke He, Mengying Bian, Samuel D. Seddon, Koushik Jagadish, Andrea Mucchietto, He Ren, Erik Kirstein, Reza Asadi, Jaeil Bai, Chao Yao, Sheng Pan, Jie‐Xiang Yu, Peter Milde, Chang Huai, Haolei Hui, Jiadong Zang, Renat Sabirianov, Xuemei M. Cheng, Guoxing Miao, Hui Xing, Yu‐Tsun Shao, Scott A. Crooker, Lukas Eng, Yanglong Hou, Jonathan P. Bird, Hao Zeng

**Affiliations:** ^1^ Department of Physics University at Buffalo The State University of New York Buffalo NY 14226 USA; ^2^ Department of Electrical Engineering University at Buffalo The State University of New York Buffalo NY 14226 USA; ^3^ School of Materials Science and Engineering Peking University Beijing 100871 China; ^4^ College of Materials Science and Engineering Beijing University of Technology Beijing 100124 China; ^5^ Institute of Applied Physics Technical University of Dresden 01187 Dresden Germany; ^6^ Mork Family Department of Chemical Engineering and Materials Science University of Southern California Los Angeles CA 90089 USA; ^7^ National High Magnetic Field Laboratory Los Alamos National Lab Los Alamos NM 87545 USA; ^8^ Department of Electrical and Computer Engineering Institute for Quantum Computing University of Waterloo Ontario N2L3G1 Canada; ^9^ Department of Physics University of Nebraska‐Omaha Omaha NE 68182 USA; ^10^ Key Laboratory of Artificial Structures and Quantum Control Shanghai Center for Complex Physics School of Physics and Astronomy Shanghai Jiao Tong University Shanghai 200240 China; ^11^ School of Physical science and Technology Soochow University Suzhou 215006 China; ^12^ Department of Physics and Astronomy University of New Hampshire Durham NH 03824 USA; ^13^ Physics Department Bryn Mawr College Bryn Mawr PA 19010 USA; ^14^ School of Materials Sun Yat‐Sen University Shenzhen 518107 China

**Keywords:** 2D magnets, anomalous Hall effect, Berry curvature, Cr2Te3, intercalation

## Abstract

Covalent 2D magnets such as Cr_2_Te_3_, which feature self‐intercalated magnetic cations located between monolayers of transition‐metal dichalcogenide material, offer a unique platform for controlling magnetic order and spin texture, enabling new potential applications for spintronic devices. Here, it is demonstrated that the unconventional anomalous Hall effect (AHE) in Cr_2_Te_3_, characterized by additional humps and dips near the coercive field in AHE hysteresis, originates from an intrinsic mechanism dictated by the self‐intercalation. This mechanism is distinctly different from previously proposed mechanisms such as topological Hall effect, or two‐channel AHE arising from spatial inhomogeneities. Crucially, multiple Weyl‐like nodes emerge in the electronic band structure due to strong spin‐orbit coupling, whose positions relative to the Fermi level is sensitively modulated by the canting angles of the self‐intercalated Cr cations. These nodes contribute strongly to the Berry curvature and AHE conductivity. This component competes with the contribution from bands that are less affected by the self‐intercalation, resulting in a sign change in AHE with temperature and the emergence of additional humps and dips. The findings provide compelling evidence for the intrinsic origin of the unconventional AHE in Cr_2_Te_3_ and further establish self‐intercalation as a control knob for engineering AHE in complex magnets.

## Graphical abstract

The unconventional anomalous Hall effect (AHE) in Cr2Te3, characterized by a sign change and humps and dips near the coercive field in its hysteresis, is attributed to an intrinsic mechanism dictated by self‐intercalated Cr atoms with spin canting, closely linked to multiple band anti‐crossings near the Fermi level.

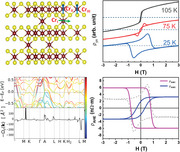

## 1 Introduction

2D magnets are fundamentally interesting because they challenge and expand our understanding of magnetic phenomena in reduced dimensions.^[^ ^1^ ^]^ Fundamental models, such as Ising, XY, and Heisenberg models can be tested in the 2D limit in these materials.^[^ ^2^ ^]^ The flexibility of integrating van der Waals (vdW) 2D magnets with other quantum materials opens up exciting possibilities for the realization of ultra‐compact spintronic and valleytronic devices,^[^ ^3^ ^]^ Ising superconductor Josephson junctions,^[^ ^4^ ^]^ and topological quantum computing devices.^[^ ^2^, ^5^ ^]^ Recently, researchers have expanded the family of 2D magnets to encompass non‐vdW materials dubbed “covalent 2D magnets”.^[^ ^6^ ^]^ A covalent 2D magnet is composed of vdW monolayers held together by covalent bonds to self‐intercalated cations located between the monolayers. Distinctly different from vdW systems, the exchange coupling, magnetic order, and spin texture in covalent 2D magnets can be controlled by the self‐intercalation, providing a new degree of freedom for manipulating their magnetism. While these materials in their bulk form such as Fe‐Se and Cr‐Te systems were reported decades ago,^[^ ^7^ ^]^ atomically thin layers of such materials have only been realized in the past decade, first by chemical synthesis^[^ ^8^ ^]^ and more recently by molecular beam epitaxy (MBE)^[^ ^9^ ^]^ and chemical vapor deposition.^[^ ^6^, ^10^ ^]^ As a prototypical covalent 2D ferromagnet, Cr_2_Te_3_ exhibits a layered structure consisting of monolayers of CrTe_2_ covalently bonded by a layer of self‐intercalated Cr atoms with ordered vacancies. It possesses strong spin‐orbit coupling (SOC) and a large perpendicular magnetic anisotropy (PMA), with a Curie temperature (*T*_C_) of ≈180 K.^[^ ^6a^ ^]^ Furthermore, the presence of spin frustration and canting arising from competing exchange interactions^[^ ^6^, ^10^ ^]^ can lead to nontrivial magnetic textures and correspondingly complex transport properties, which can be leveraged for classical and quantum information applications.^[^ ^2^, ^5^ ^]^

The anomalous Hall effect (AHE) is a phenomenon where a transverse Hall voltage is generated in the absence of an external magnetic field. It is typically observed in ferromagnetic, ferrimagnetic, and even some non‐collinear antiferromagnetic materials where the specific arrangement of spins breaks time‐reversal symmetry in combination with particular lattice symmetries.^[^ ^11^ ^]^ The origin of the AHE can be attributed to three primary mechanisms: the intrinsic mechanism related to Berry curvature in the momentum space, and extrinsic mechanism involving side jump or skew scattering.^[^ ^11^ ^]^ In a range of materials with complex spin structures such as SrRuO_3_^[^ ^12^ ^]^ and Cr‐Te systems,^[^ ^13^ ^]^ unconventional AHE hysteresis loops, marked by additional humps and dips, have been observed and are often attributed to the topological Hall effect (THE). THE is often believed to arise from chiral spin textures such as skyrmions, and typically scales linearly with the 2D density of topological charges, contributing to non‐zero Berry curvature in real space.^[^ ^14^ ^]^ However, these interpretations have rarely been substantiated by magnetic force microscopy (MFM) or Lorentz transmission electron microscopy (LTEM) measurements. Even in the case where skyrmionics bubbles were observed by LTEM,^[^ ^15^ ^]^ the density of the topological charges often does not match with the amplitude of the anomalous Hall conductivity. Alternatively, the unconventional behavior has also been attributed to the superposition of two AHE signals with opposite signs and different coercive fields, contributed by different regions of the sample having distinct magnetic properties,^[^ ^9^, ^16^ ^]^ such as those arising from spatial inhomogeneities (“two‐channel AHE”). Nonetheless, the magnetization hysteresis often does not match with the decomposed AHE signal.^[^ ^12b^ ^]^ Thus, unraveling the unconventional AHE in complex spin systems remains a formidable challenge, impeding the envisioned device applications in such materials.

In this work, we unravel the mystery of the unconventional AHE observed in MBE grown Cr_2_Te_3_ 2D films. Combining temperature‐dependent transport studies, including the Hall effect and magneto‐resistivity (MR) measurements, with MFM and magnetization and magneto‐optical measurements, we unambiguously rule out both THE and two‐channel AHE arising from spatial inhomogeneities, such as those induced by interfacial strain, as the underlying cause of the unconventional AHE behavior. We show instead that the behavior is intrinsic to the electronic structure of Cr_2_Te_3_, and is closely associated with the spin texture of the self‐intercalated Cr cations. The Weyl‐like nodes created by the SOC‐induced gap opening near the Fermi level play a pivotal role in momentum space Berry curvature and unconventional AHE conductivity. With changing temperature, the band dispersion and electron occupation are sensitively modulated by the changing spin canting angle of the self‐intercalated Cr due to thermal fluctuations, shifting the position of the Fermi level relative to these nodes. The contribution to the Berry curvature due to the Weyl‐like nodes is opposite to that from bands without anti‐crossing, and the two contributions exhibit an antagonistic temperature dependence. Consequently, the superposition of two AHE signals with opposite signs and distinct field dependences give rise to the sign change of AHE resistivity with temperature, as well as to additional humps and dips in the AHE hysteresis. We suggest, therefore, that the AHE can be a sensitive probe for hidden magnetic orders in systems with complex spin structures, which would otherwise elude detection by conventional probes such as magnetometry. Furthermore, we propose that the Berry curvature and AHE can be manipulated by self‐intercalation in covalent 2D magnets, e.g. by changing its chemical order, which can be harnessed for quantum device applications.

## 2 Results and Discussion

**Figure 1:**
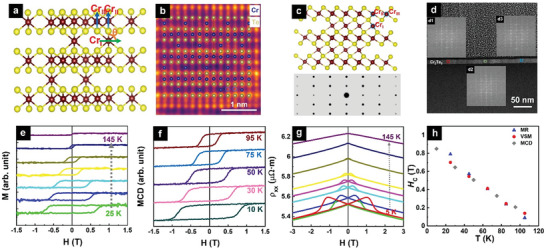
Structural and magnetization characterizations and MR measurements of the Cr_2_Te_3_ thin film. a) A schematic of the atomic structure of Cr_2_Te_3_, viewed along [100] zone axis, where Cr_I_, Cr_II_, and Cr_III_ are three inequivalent Cr sites. b) A cross‐sectional HAADF‐STEM image of the Cr_2_Te_3_ thin film taken along the [100] axis with iDPC technique, consistent with the atomic structure model. The blue circles mark the atomic columns with a weak contrast, indicating partially occupied vacancy sites due to chemical disorder. c) A schematic of the atomic structure (top) and simulated diffraction pattern (bottom) obtained from the atomic model of Cr_2_Te_3_, viewed along [210] zone axis. d) A cross‐sectional HAADF‐STEM image of Cr_2_Te_3_ thin film taken along the [210] zone axis. Inset: (d1–d3) corresponding FFT patterns of the square‐colored areas indicated in the HAADF‐STEM image in (d), matching the simulated electron diffraction pattern in (c). e) Out‐of‐plane magnetic hysteresis loops measured at different temperatures. (From bottom to top, measurement temperatures are 25, 45, 65, 85, 105, 125, and 145 K, respectively.) f) Out‐of‐plane MCD hysteresis loops measured at different temperatures (From bottom to top, measurement temperatures are 10, 30, 50, 75, and 95 K, respectively) using 700 nm light. g) Temperature‐dependence of longitudinal resistivity ρ_*xx*_ as a function of the out‐of‐plane magnetic field. (From bottom to top, measurement temperatures are 5, 25, 45, 65, 75, 85, 105, 125, and 145 K, respectively.) h) *H*_C_ as a function of temperature extracted from magnetic (circle), MCD (square), and MR (triangle) hysteresis measurements.

**Figure** **1**a shows a schematic of the atomic model of the covalent 2D magnet Cr_2_Te_3_ with a hexagonal structure and a P $\bar{3}$ 1c (No. 163) space group,^[^ ^6^, ^9^ ^]^ viewed along the [100] zone axis. A single unit cell consists of a CrTe_2_ bilayer connected by 1 intercalated Cr cation per 3 Te‐Cr‐Te blocks (denoted by Cr_I_). The X‐ray diffraction (XRD) pattern (Figure S1a, Supporting Information) of the MBE Cr_2_Te_3_ film confirms the expected hexagonal crystal structure with (001) orientation. The atomic structure was further characterized by high‐angle annular dark‐field aberration‐corrected scanning transmission electron microscopy (HAADF‐STEM) combined with integrated differential‐phase contrast (iDPC) imaging technique, as shown in the cross‐sectional image of a Cr_2_Te_3_ film of a thickness of 8‐unit cell (≈10 nm) in Figure 1b, viewed along [100] axis, which matches well with the atomic model in Figure 1a. It should be emphasized that in a nominally single‐crystalline sample grown by MBE, a small chemical disorder inevitably exists, leading to a small fraction of the vacancy sites being occupied. This is shown by the atomic columns with a weak contrast marked by the blue circles in Figure 1b. The single‐crystalline nature of the film is further evidenced by the identical fast Fourier transform (FFT) patterns taken at different spots (Figure 1d1–d3) of the cross‐sectional HAADF‐STEM image (atomic STEM images taken at these spots are shown in Figure S1g–i, Supporting Information), which is consistent with the simulated diffraction pattern obtained from the atomic model of Cr_2_Te_3_ viewed along [210] zone axis (Figure 1c).

The basic magnetic properties were characterized by the magnetization and magnetic circular dichroism (MCD) hysteresis loops, together with magneto‐resistivity (MR) measurements. The Cr_2_Te_3_ film exhibits an out‐of‐plane easy axis, as shown by the square hysteresis loops with a full remanence in Figure 1e (magnetization was measured in the out‐of‐plane direction at temperatures below *T*_C_ ranging from 25–145 K) and in Figure 1f measured by MCD. This is consistent with the expected large magnetic anisotropy constant of ≈1 × 10^6^ J m^−3^ for Cr_2_Te_3_.^[^ ^6^, ^8^, ^10^ ^]^ A closer inspection of Figure 1e,f reveals that all hysteresis loops exhibit a single‐phase behavior, with no discernable steps. The single‐phase behavior is further confirmed by the corresponding longitudinal MR results in Figure 1g, where the applied field is out of plane. The MR curves show the typical butterfly shape, ubiquitous for many magnetic systems,^[^ ^17^ ^]^ and is a manifestation of the magnetic hysteresis. The coercivity (*H*_C_) values extracted from the MR hysteresis match closely with those from the magnetic and MCD hysteresis, as seen in Figure 1h. The atomic structural, magnetization, MCD, and MR measurements suggest that our Cr_2_Te_3_ film is single‐crystalline and magnetically homogeneous with no detectable secondary phase.

**Figure 2:**
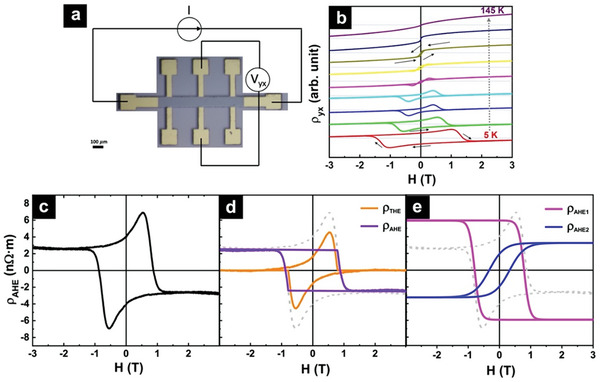
Hall effect measurements and two interpretations of the observed unconventional AHE. a) A schematic of the Hall bar device used for Hall effect measurements. b) Shown from bottom to top are the magnetic field‐dependent Hall resistivity ρ_*yx*_ measured at temperatures of 5, 25, 45, 65, 75, 85, 105, 125, and 145 K, respectively. The solid arrows indicate the looping directions, showing a change of polarity of the measured Hall resistivity with changing temperature. c) A representative ρ_*AHE*_ loop measured at 25 K, obtained by subtracting the OHE contribution. d) Fitting by an AHE loop and a THE loop, and e) fitting by two AHE loops to reproduce the observed humps and dips in the AHE loop in (c).

We next focus on the AHE in the Cr_2_Te_3_ film, which exhibits unconventional behaviors. The magnetic‐field‐dependent Hall resistivity (ρ_*yx*_) taken at different temperatures are presented in **Figure** **2**b. In the Hall measurements, a fixed current of 100 µA was passed between longitudinal probes, and the Hall voltage was measured between transverse probes, as depicted in Figure 2a. In general, in a homogeneous ferromagnet below *T*_C_, the AHE resistivity is linearly proportional to the out‐of‐plane magnetization, and therefore the shape of its hysteresis should mimic that of the magnetic hysteresis loops. However, as seen from Figure 2b, two unconventional behaviors are observed. First, there is a sign change of the Hall resistivity as a function of temperature: ρ_*yx*_ at high fields at which the magnetization saturates is initially positive at high temperatures close to *T*_C_. Upon decreasing temperature, its magnitude decreases. At ≈50 K, it crosses zero and becomes negative at low temperatures. Second, prominent humps and dips are observed at fields near *H*_C_, at temperatures below ≈100 K. Such humps and dips in AHE hysteresis have been frequently attributed to the THE, which is considered as evidence for the presence of chiral spin textures such as magnetic skyrmions.^[^ ^13a–c^ ^]^ However, these features have also been interpreted as the coexistence of two AHE signals with opposite polarities.^[^ ^9^, ^16^ ^]^ It is difficult to discern the two interpretations from AHE measurements alone. For example, the AHE resistivity (ρ_*AHE*_) loop at 25 K, obtained by subtracting the linear ordinary Hall effect (OHE) background (see Figure 2c), has been decomposed into an AHE and a THE in Figure 2d and two AHE loops with opposite polarities in Figure 2e, respectively (for more details of the decomposition, please refer to Section S1, Supporting Information). Herein, ρ_*AHE*1_ (pink) has a negative polarity, i.e., ρ_*AHE*1_ is negative at positive saturation field; while ρ_*AHE*2_ (blue) has a positive polarity, i.e., ρ_*AHE*2_ is positive at positive saturation field. The superposition of the two signals using both methods reproduce the measured AHE loop, as can be seen in Figure 2d,e.

**Figure 3:**
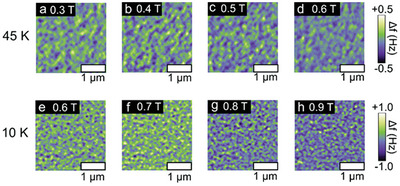
MFM images measured at representative temperatures of 45 and 10 K with different magnetic fields. Evolution of magnetic domains of the sample measured at 45 K with field values of a) 0.3 T, b) 0.4 T, c) 0.5 T, and d) 0.6 T; and at 10 K with field values of e) 0.6 T, f) 0.7 T, and g) 0.8 T and h) 0.9 T.

To determine the origin of the humps and dips in ρ_*AHE*_ hysteresis, we first imaged the magnetic domain structures of Cr_2_Te_3_ using MFM, at 45 and 10 K, where the unconventional behaviors are evident. The sample's magnetization was initially saturated at ‐2 T, after which the field was scanned to 2 T and then back to ‐2 T. **Figure** **3** shows selected MFM images acquired at magnetic fields close to where the humps and dips emerge. The magnetic contrast comes from the frequency shift (Δ*f*) of the resonating cantilever, caused only by the magnetic interactions between the cantilever and the sample surface's magnetic texture. At 45 K, the magnetic domain patterns exhibit alternating spin‐down (positive frequency shift; green) and spin‐up (negative frequency shift; blue) stripe‐like domains, consistent with the strong PMA of the Cr_2_Te_3_ film. As the field is scanned progressively toward more positive (negative) values, the fraction of spin‐up (spin‐down) domains increases. This occurs through a nearly stochastic flipping of the domains, keeping the characteristic dimensions of the domains relatively constant, as opposed to the nucleation of domains followed by domain wall propagation. It appears that domain walls are locally pinned, and increasing the field magnitude only leads to the reversal of more domains. With decreasing temperature from 45 to 10 K, the width of the domains decreases substantially due to the larger magnetic anisotropy.^[^ ^18^ ^]^ Nevertheless, the stochastic domain flipping behavior remains unchanged. The observation of stripe‐like domains and their relatively independent reversal is markedly different from that expected from a phase transition into a skyrmion‐like spin texture, thereby ruling out THE as the origin of the unconventional AHE behavior.

**Figure 4:**
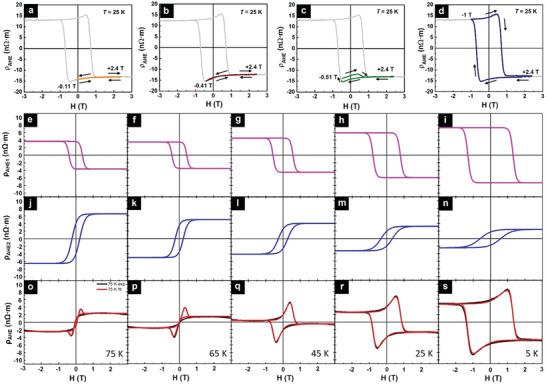
Minor ρ_*AHE*_ loops and fitting results of the temperature‐dependent ρ_*AHE*_ loops for the unannealed samples. Minor ρ_*AHE*_ loops measured at 25 K for different stopping fields a) −0.11 T, b) −0.41 T, c) −0.51 T, and d) −1 T (Gray lines are corresponding full AHE loops for comparison). e–i) ρ_*AHE*1_ and j–n) ρ_*AHE*2_ loops with opposite polarities that are used to fit ρ_*AHE*_ loops, respectively. o–s) Corresponding fitting results (red) and measured (black) ρ_*AHE*_ loops. From left to right, the measuring temperature decreases from 75 to 5 K.

Minor AHE resistivity loops measured at 25 K are used to further elucidate the origin of the unconventional AHE behavior. Minor AHE loops have been used previously to distinguish THE from two‐channel AHE behavior in MnBi_2_Te_4_,^[^ ^16b^ ^]^ SrRuO_3_^[^ ^16d^ ^]^ and Cr_2_Te_3_.^[^ ^9^, ^16^ ^]^ Here the minor loops were obtained by first saturating the sample at a field of +2.4 T, followed by sweeping the fields to successively smaller negative values (stopping fields) ranging from ‐2.4 to ‐0.11 T, and then back to +2.4 T. As can be seen from **Figure** **4**a,b, at a stopping field of ‐0.41 T and below, no hump or dip is observed. As the field reaches ‐0.51 T (Figure 4c), both a dip and a hump are observed, but with uneven amplitudes. Only at the field of ‐1 T (Figure 4d) does the AHE hysteresis exhibit symmetric hump and dip consistent with those of the major loop (see results at other stopping fields in Figure S3, Supporting Information). Such a behavior is inconsistent with THE, since the topological spin texture should be robust and independent of field history. Instead, it can be understood as originating from the superposition of two AHE resistivity loops with opposite polarities and different coercivity *H*_C_, denoted as ρ_*AHE*1_ (negative) and ρ_*AHE*2_ (positive) that have been defined in Figure 2e. In this scenario, at ‐0.11 T (Figure 4a), both ρ_*AHE*1_ and ρ_*AHE*2_ remain unswitched since their coercive fields have not been reached, yielding to the absence of hysteresis. At ‐0.41 T (Figure 4b), only the magnetically soft component with lower *H*_C_ is partially reversed and the hard component is hysteresis‐free, resulting in a minor loop dominated by that of ρ_*AHE*2_, without hump or dip. Once the field reaches ‐0.51 T (Figure 4c), a partial switching of ρ_*AHE*1_ with an opposite sign to that of ρ_*AHE*2_ develops, giving rise to a hump in the positive field branch at a smaller amplitude than that of the dip in the negative field branch. By increasing the field to ‐1 T, above the *H*_C_ of the hard component, the hump feature is fully developed in the positive field branch, collapsing the minor loop onto the corresponding major loop (Figure 4d). Combined with MFM results, these findings definitively rule out THE as the source of the unconventional AHE behavior and affirm that the humps and dips observed in ρ_*AHE*_ arises from two AHE loops with opposite signs (but not from spatial inhomogeneity, as well be discussed below).

In Figure 4o–s, the total AHE resistivity hysteresis observed at different temperatures are fitted using two AHE resistivity loops with opposite signs: ρ_*AHE*1_ as shown in Figure 4e–i is negative and ρ_*AHE*2_ as shown in Figure 4j–n is positive. It can be seen clearly that the measured total ρ_*AHE*_ (black) are well‐fitted by the superposition (red) of ρ_*AHE*1_ and ρ_*AHE*2_ loops. Crucially, it is found that the *H*_C_ of the ρ_*AHE*1_ hysteresis match well with those obtained from magnetic and MR hysteresis loops (see Figure S4, Supporting Information), suggesting that the AHE1 signal is associated with the magnetization of Cr_2_Te_3_. The emergence of humps and dips in the total Hall resistivity curve is due to the different *H*_C_ values of ρ_*AHE*1_ and ρ_*AHE*2_ hysteresis with opposite signs. Furthermore, the temperature dependence of ρ_*AHE*1_ and ρ_*AHE*2_ hysteresis exhibits antagonistic trends: while the magnitude of ρ_*AHE*1_ increases with decreasing temperature, that of ρ_*AHE*2_ decreases with decreasing temperature. At the critical temperature of ≈45 K, a change in polarity in total ρ_*AHE*_ is observed. Neither the *H*_C_ values nor the temperature dependence of its magnitude matches that of bulk magnetization, suggesting that ρ_*AHE*2_ has a different origin.

The emergence of two AHE channels in CrTe*_x_* has been ascribed to spatial inhomogeneities, such as thickness variations, defects, and interface modulation.^[^ ^9^, ^16^ ^]^ However, in our case, both cross‐sectional TEM and AFM images showed surface roughness of our film to be ≈0.2 nm (see Figures 1 and S1b, Supporting Information), making it unlikely for thickness variation to contribute to a second phase. In a typical ferromagnet, the field dependence of ρ_*AHE*_ matches with the magnetic hysteresis.^[^ ^11^, ^19^ ^]^ However, as discussed earlier, both the magnetization and MR measurement results show single‐phase behavior, with *H*_C_ values match closely with those derived from ρ_*AHE*1_ loops. No evidence of a second phase responsible for ρ_*AHE*2_ exists in either magnetization or MR hysteresis. Additionally, MFM also revealed uniform stripe‐like domains, absence of a secondary phase with different magnetic parameters. These observations unambiguously rule out spatial inhomogeneities, e.g., thickness variations as the source of ρ_*AHE*2_. Another potential source of a secondary phase might arise from modification of the magnetic properties within the interfacial region due to strain induced by the substrate.^[^ ^20^ ^]^ Given the atomic thinness of the interface, it could elude detection through bulk magnetic measurements. However, the increase in the magnitude of ρ_*AHE*2_ with increasing temperature suggests that it cannot be trivially related to magnetization; otherwise it would decrease with increasing temperature. To further rule out interfacial origin of ρ_*AHE*2_, we performed two additional experiments. First, we measured and resolved the two AHE components for MBE films with two different thicknesses: 8‐unit cell and 50‐unit cell. If AHE1 is dominated by bulk contribution (as it scales with bulk magnetization) while AHE2 is contributed by a secondary phase derived from interfacial strain, the magnitude of AHE1 is expected to depend strongly on film thickness while AHE2 should be insensitive to it. However, as shown in Figure S6a,b (Supporting Information), the Hall resistance for AHE1 (*R*_AHE1_) and AHE2 (*R*_AHE2_) measured at different temperatures have the same order of magnitude for the same film thicknesses, while both *R*_AHE1_ and *R*_AHE2_ of the 8‐unit cell sample are 3–4 orders of magnitude higher than those of the 50‐unit cell sample. This strongly suggests that both AHE1 and AHE2 originate from the bulk of the film rather than the interface.

**Figure 5:**
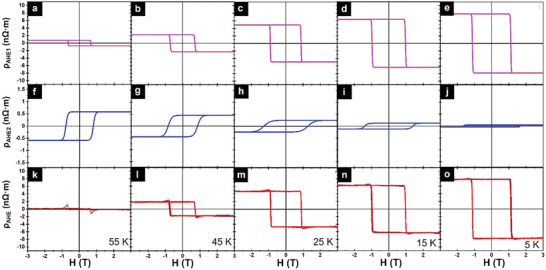
Temperature‐dependent Hall resistivity for the annealed samples. a–e) Magenta and f–j) blue curves represent ρ_*AHE*1_ and ρ_*AHE*2_ loops with different polarities that are used in the fittings, respectively. k–o) The corresponding fitting results (red) for the measured (black) total AHE resistivity ρ_*AHE*_ loops at different temperatures. From left to right, the temperature decreases from 55 to 5 K.

Next, we subjected the as‐grown sample to ultrahigh‐vacuum (UHV) annealing, at the same growth temperature of 350 °C for 30 minutes. Such a moderate heat treatment is not expected to modify the interfacial strain, which is determined by the lattice mismatch between the Cr_2_Te_3_ film and sapphire substrate; nor should it change the phase and composition of the Cr_2_Te_3_ film, due to the moderate annealing temperature and the presence of an Al_2_O_3_ capping layer. This is further verified by the fact that there is no significant change in the Curie temperature of the film before and after annealing (see Figure S8, Supporting Information).^[^ ^21^ ^]^ Surprisingly, the annealing resulted in dramatic changes in the AHE behavior, as seen in **Figure** **5**. While the humps and dips persisted, they appear as sharp spikes. Strikingly, the humps and dips shifted from the first and third quadrants in the as‐grown sample to the second and fourth quadrants. These changes cannot be explained by two‐channel AHE resulting from interfacial strain. Furthermore, in previous reports, such unconventional AHE have been observed in films on various substrates including SrTiO_3_,^[^ ^13a^ ^]^ Al_2_O_3_,^[^ ^7b^ ^]^ BN^[^ ^13b^ ^]^ and topological insulator Bi_2_Te_3_,^[^ ^13e^ ^]^ as well as in free‐standing single crystals.^[^ ^13c^ ^]^ These decisively rule out the interface as the origin of the two‐channel AHE behavior. Given that the dominating structural change post‐annealing is the enhanced chemical ordering of the Cr_I_ sites, we propose that the observed unconventional AHE is intrinsic to Cr_2_Te_3_, with the self‐intercalated Cr playing a central role.

This naturally raises the question on how the Cr_I_ sublattice contributes to the AHE while eluding detection by magnetization and MR measurements. Earlier neutron diffraction studies on single crystal Cr_2_Te_3_ showed that the intercalated Cr_I_ exhibits a tiny magnetic moment, ≈−0.14 µ_B_ versus 2.78 and 2.52 µ_B_ for Cr_II_ and Cr_III_ in the CrTe_2_ layer.^[^ ^22^ ^]^ This suggests that the self‐intercalated Cr_I_ moments are canted and lie nearly in the plane, with a small z‐component antiferromagnetically aligned with those of Cr_II_ and Cr_III_. In other words, the canting angle (θ) is slightly larger than 90°, where θ is defined as the angle between the Cr_I_ moment and the +z direction, as shown schematically in Figure 1a. Our magnetization measurement of the film (Figure S7 and Text S2, Supporting Information) yields an estimated value of ≈15.5 ± 1.0 µ_B_ per unit cell, consistent with 15.6 µ_B_/unit cell from the neutron scattering studies,^[^ ^22^ ^]^ but is substantially smaller than ≈3 µ_B_/Cr expected for a ferromagnetic configuration, confirming spin canting. Since the atomic fraction of Cr_I_ is 25% in Cr_2_Te_3_, the contribution of Cr_I_ to the total magnetization is ≈1%. It is thus not surprising that out‐of‐plane magnetization and MR measurements failed to detect Cr_I_ contribution. The strong canting of the self‐intercalated Cr moments is a result of the competing antiferromagnetic (AFM) exchange coupling between Cr_I_ with its nearest neighbor (NN) Cr_II_ and ferromagnetic (FM) exchange coupling with its next nearest neighbor (NNN) Cr_III_.^[^ ^6a^ ^]^ Such competition also weakens the exchange coupling of the Cr_I_ sublattice to the CrTe_2_ layer, which can cause it to reverse its magnetization at *H*_C_ different from that of the CrTe_2_ layer. Nevertheless, as will be explained below, the hidden magnetic order of Cr_I_ significantly contributes to the momentum space Berry curvature, giving rise to intrinsic AHE contributions,^[^ ^11^ ^]^ a point that is further confirmed by the fact that ρ_*AHE*_ scales linearly with $\rho_{xx}^{2}$ (see Figure S6c, Supporting Information).

**Figure 6:**
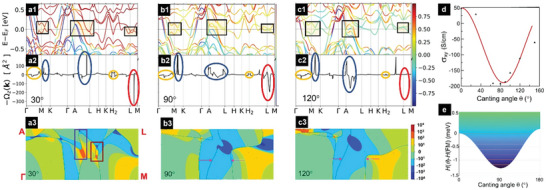
Calculated band structure, Berry curvature, canting angle‐dependent energy and σ_*xy*_. The band structure of Cr_2_Te_3_ with Cr_I_ moment canting angle θ of a1) 30°, b1) 90°, and c1) 120°. The bands are color‐coded according to their spin projection. The corresponding Berry curvatures in high‐symmetry directions of the Brillouin zone for θ of a2) 30°, b2) 90°, and c2) 120°. The surface contour plot of the Berry curvature in the Γ‐M‐L‐A plane for θ of a3) 30°, b3) 90°, and c3) 120°. The color scale represents the sign and magnitude of the Berry curvature. d) σ_*xy*_ as a function of θ; the red line is the guide to the eye. e) The difference in Heisenberg energies between the canting angle θ and the FM configuration (*θ* = 0), as a function of θ.

To understand the intrinsic two‐channel AHE behavior in Cr_2_Te_3_ and its temperature dependence, we preformed first principles calculations of the band structure and Berry curvature^[^Ω_*z*_(*k*)] for different canting angles (θ) of Cr_I_ moment. In **Figure** **6**, the top panel illustrates the calculated band structures of Cr_2_Te_3_ for θ of 30° (a1), 90° (b1) and 120° (c1), respectively (see result for the ferromagnetic configuration θ = 0 in Figure S10, Supporting Information), where the bands are color‐coded according to their spin projection. The middle panel a2‐c2 shows the corresponding ‐Ω_*z*_(*k*)^[^‐Ω_*z*_(*k*) is plotted as it has the same sign as σ_*xy*_ and ρ_*yx*_] in high‐symmetry directions of the Brillouin zone (BZ). The Berry curvature is further illustrated in the 2D contour plots in Figure 6a3–c3 in the bottom panel.

Interestingly, as seen in Figure 6a1–c1, the spin characters of the bands near the Fermi level change significantly with *θ*, suggesting that these bands are strongly hybridized with Cr_I_ states. A striking feature is the multiple band anti‐crossings due to the strong SOC, as marked by the black boxes in Figure 6a1–c1. These spin‐polarized anti‐crossing bands resemble Weyl nodes^[^ ^23^ ^]^ with nearly linear dispersion. Remarkably, the position of the Fermi level relative to these Weyl‐like nodes is extremely sensitive to *θ*. This sensitivity arises because the canting of Cr_I_ moments enhances the mixing of spin‐up and spin‐down states, strongly modifying the dispersion of these bands and their relative electron occupation. When the Fermi level crosses these nodes, they act as sources or sinks of Berry curvature,^[^ ^24^ ^]^ leading to prominent positive peaks (e.g., those around M‐K, A, and A‐L points marked by blue ovals) and negative peaks (e.g., the one around L‐M points marked by the red oval), as seen in Figure 6a2–c2. As shown in the 2D contour plots in Figure 6a3–c3, when the Fermi surface breaks up into disconnected sheets, such as those located at the Γ, M, and L corners of the BZ, the contribution of the states enclosed by the Fermi surface to the Berry curvature is very small. In contrast, when the Fermi surface sheets form nodes due to band anti‐crossing, the Berry curvature develops large peaks.

Next, we examine the details of the evolution of band structure and Berry curvature as a function of *θ*. For example, consider the SOC‐split bands around the L‐M points shown by the black boxes in Figure 6a1–c1. At *θ* = 30°, they are located below the Fermi energy (Figure 6a1). As a result, the Berry curvature is small, as shown by the sharp spike with negligible area marked by the red oval in Figure 6a2. Correspondingly, as marked by the boxes in Figure 6a3, we observe “dipole” pockets with alternating positive and negative Berry curvature peaks in the 2D plot. As *θ* increases to 90° (close to the ground state canting angle), the anti‐crossing bands shift upward so that the Fermi energy falls within the SOC‐split gap (see Figure 6b1), substantially increasing their contribution to the Berry curvature, as evidenced by the negative peak with a large area (Figure 6b2). Accordingly, the Fermi surface sheets in the 2D plot (Figure 6b3) shift apart. This results in a reduction in the intensity but an increase in the area of the negative peaks. The two‐node pockets shown by the boxes in Figure 6a3 vanish as a result of a sign switching of the positive region in Figure 6a3 to negative in Figure 6b3. The overall Berry curvature is strongly negative at *θ* = 90°. With *θ* increasing further to 120°, the anti‐crossing bands shift above the Fermi energy (Figure 6c1), diminishing their contribution to the Berry curvature (Figure 6c2). In Figure 6c3, the Fermi surface sheets separate further apart, reducing the negative contribution. Meanwhile, a new Fermi surface node appears, leading to Berry curvature peaks dominated by the positive ones near the A point of the BZ. This makes the overall contribution less negative. Thus, the variation of Berry curvature with the canting angle is nonmonotonic, being the most negative at *θ* = 90°. There are also bands crossing the Fermi level without Weyl‐like nodes, as seen in Figure 6a1–c1. The Berry curvature resulting from these bands changes relatively slowly with θ, as marked by the orange ovals in Figure 6a2–c2. The extreme sensitivity of the anti‐crossing band positions (Weyl‐like nodes) relative to the Fermi level to the canting angle of the self‐intercalated Cr cations is a unique feature in covalent 2D magnets, differentiating them from pure vdW magnets. This makes the Berry curvature and AHE highly tunable.

The AHE conductivity (σ_*xy*_) is obtained by integrating the Berry curvature over the BZ, $\sigma_{xy}=-\frac{e^{2}}{\hbar }\int_{BZ}\frac{d^{3}k}{{\left(2\pi \right)}^{3}}\Omega_{z}\left(k\right)$. Experimentally, ρ_*yx*_ is measured and related to the conductivity as $\rho_{yx}\approx \frac{\sigma_{xy}}{\sigma_{xx}^{2}}$, obtained by inverting the conductivity tensor in the approximation that σ_*xy*_ ≪ σ_*xx*_. The canting angle‐dependent σ_*xy*_ is plotted in Figure 6d. σ_*xy*_ is negative and exhibits a minimum at the ground state canting angle of ≈90°. It increases with both increasing and decreasing θ, which suggests that any deviation from ≈90° will lead to an increase in σ_*xy*_. We note that the magnitude of the calculated σ_*xy*_ is much larger than the experimentally measured ones. Factors such as different magnetic textures of the self‐intercalated Cr and variations in vacancy concentration and ordering can contribute to this discrepancy. However, this should not affect the overall trend of the angular dependence of σ_*xy*_.

With the above discussions, we are ready to understand the humps and dips observed in the ρ_*AHE*_ hysteresis loops and their temperature dependence. ρ_*AHE*_ measured experimentally can be decomposed into two components, due to the weakened coupling of the Cr_I_ sublattice to CrTe_2_ layers as mentioned earlier. ρ_*AHE*1_ is negative, and decreases in magnitude with increasing temperature, as expected from temperature‐dependent magnetization of a typical ferromagnet. ρ_*AHE*1_ originates from bands crossing the Fermi level lacking Weyl‐like nodes and is thus nearly independent of θ. ρ_*AHE*2_, on the other hand, is positive and increases with increasing temperature, and thus does not scale with magnetization. This is because they are associated with bands exhibiting avoided crossing and highly sensitive to θ, and is thus dictated by the changing θ with temperature. At low temperatures, the superposition of large negative ρ_*AHE*1_ and small positive ρ_*AHE*2_ leads to an overall negative ρ_*AHE*_, as seen in Figure 4. As temperature increases, thermal fluctuation causes the deviation of θ from its ground state value of ≈90°. As can be seen from the energy profile in Figure 6e, the minimum of the energy for the canted configuration is ≈1.3 meV below that of the FM state. This is ≈10 times smaller than the onsite exchange coupling parameters. Thus, increasing the temperature will cause the spin moment of Cr_I_ to disorder. A broad range of canting angles will be thermally accessible at relatively low temperatures of a tenth of *T_C_* (cut out schematically shown by the horizontal lines). 〈σ_*xy*_〉 is then obtained by averaging all accessible angles, given by $\left\langle \sigma_{xy}\right\rangle =\int_{\theta }d\theta \;\sigma_{xy}\left(\theta \right)\rho \left(\theta ,T\right)$, where ρ(θ) is an angular distribution function. Consequently, 〈σ_*xy*_〉 and ρ_*AHE*_ increase and eventually switch sign to become positive at sufficiently high temperatures.

Furthermore, while the picture presented above is consistent with experimental observations in the as‐deposited film, evidence is even more compelling from the AHE results of the annealed sample shown in Figure 5. Annealing enhances the chemical ordering of the Cr_I_ sublattice, leading to squarer AHE hysteresis loops. Further, the previously observed humps and dips transform into sharp spikes. The chemical order further increases the MAE of the Cr_I_ sublattice, resulting in a larger *H*_C_ for ρ_*AHE*2_ hysteresis compared to ρ_*AHE*1_. Consequently, the spikes now emerge in the second and fourth quadrants instead of in the first and third quadrants for the unannealed sample. Neither the emergence of sharp spikes nor the shift in their positions can be explained by THE or two AHE from spatial inhomogeneities. These results further suggest a viable approach to tuning the Berry curvature by controlling the ordering of self‐intercalated Cr and vacancies, thus allowing for the control of AHE conductivity.

## 3 Conclusion

In conclusion, the unconventional AHE in 2D covalent magnet Cr₂Te₃ with self‐intercalated Cr cations, characterized by humps and dips and the temperature‐dependent sign change in AHE hysteresis, originate from an intrinsic mechanism driven by self‐intercalated Cr cations with large spin canting angles that change with thermal fluctuations, which contrasts with previously proposed explanations such as the THE and two‐channel AHE induced by spatial inhomogeneities. This occurs because the canting of self‐intercalated Cr moments enhances the mixing of the spin‐up and spin‐down states and strongly modifies the dispersion of the electronic bands with multiple Weyl‐like nodes, sensitively modulating the positions of these bands with respect to the Fermi level and altering their contribution to the Berry curvature and thus AHE resistivity. The canted Cr spin sublattice evades detection by magnetization and MR measurements but is sensitively detected by AHE. These findings provide evidence for the intrinsic origin of the unconventional AHE in Cr₂Te₃, underscoring the role of self‐intercalation as a pivotal control mechanism. We further propose that the Berry curvature and AHE conductivity can be manipulated by the chemical ordering in covalent 2D magnets, opening new avenues for spintronic device applications.

## 4 Experimental Section

### Sample Growth

The growth of Cr_2_Te_3_ thin films was carried out in a molecular beam epitaxy (MBE) system under an UHV environment of 10^−10^–10^−9 ^Torr. Cr_2_Te_3_ thin films were grown on (0001) sapphire substrate by co‐deposition from Cr and Te sources in an MBE chamber. The film thickness was controlled by the deposition time as calibrated from X‐ray reflectometry. To protect the thin films from oxidation during characterizations, a capping layer of 5 nm Al_2_O_3_/5 nm Pt was deposited. Insulating Al_2_O_3_ (0001) was used as substrates, whose surface quality was insured by ex situ chemical and thermal cleaning and in situ outgassing at 800 °C for 30 minutes. Film thicknesses were tuned from 3 to 120 nm. Selected samples were also annealed in UHV at 300 °C.

### Magnetization and Transport Measurements

Magnetic hysteresis loops and MR were measured in the temperature range of 4–300 K in a Quantum Design Physical Property Measurement System (PPMS) equipped with a 7 T super‐conducting magnet. For electrical transport measurements, samples were fabricated into Hall‐bar patterns with a standard photolithography and subsequent reactive ion etching (RIE). A fixed current of 100 µA was passed between longitudinal probes, and the Hall voltage was measured between transverse probes.

### MCD

Broadband MCD spectroscopy was performed in both reflection and transmission geometries using wavelength‐tunable narrowband light derived from a xenon white light source filtered through a 300 mm spectrometer. The probe light was intensity modulated by a mechanical chopper, and then modulated between right and left circular polarizations by a linear polarizer and photoelastic modulator. The light was focused on the sample, and back‐reflected (or transmitted) light was detected by an avalanche photodiode detector. The signal was demodulated by two lock‐in amplifiers, referenced to the chopper and photoelastic modulator frequencies (137 Hz and 50 kHz, respectively). MCD is given by the normalized difference between the right and left circularly polarized detected intensities, (I_R_ − I_L_)/(I_R_ + I_L_).

### MFM

In order to explore the magnetic nanostructure potentially responsible for the features in the Hall resistivity measurements, MFM data were acquired on an UHV environment (base pressure below 2 × 10^−10^ mbar), low temperature scanning force microscope, capable of measuring at a temperature range of 4–300 K and applying an axial (surface normal) applied magnetic field. The surface was initially planarized with topographic scanning before a 20 nm lifted hovering MFM mode was conducted.

### Cross‐Sectional STEM Sample Preparation

The cross‐section STEM sample of the Al_2_O_3_/Cr_2_Te_3_ film grown on sapphire substrate was prepared by using Focused Ion Beam (FIB) milling. It was thinned down to 70 nm thick at an accelerating voltage of 30 kV with a decreasing current from 0.79 nA to 80 pA, followed by a fine polish at an accelerating voltage of 2 kV with a small current of 21 pA to remove the amorphous layer.

### HAADF‐STEM Characterization

The atomically resolved HAADF‐STEM images were carried out on an aberration‐corrected scanning transmission electron microscope (FEI Tian Themis 60–300 kV, operating at 300 kV). A screening current of ≈0.05 nA was used to obtain HAADF images. The iDPC imaging was also used, which measures the projected electrostatic potential instead of the integrated scattering signal of the atomic column.

### XRD Spectrum

To elucidate the crystal structure and crystallinity of Cr_2_Te_3_ films, X‐ray analysis has been carried out. X‐ray diffraction was performed using Malvern Panalytical Empyrean diffractometer at a voltage of 45 kV and current of 40 mA with CuKα (λ = 1.54059 Å) radiation.

### Density Functional Theory (DFT)‐Based Ab‐Initio Calculations

DFT‐based ab initio calculations were performed by using the Vienna ab initio Simulation Package. The Perdew–Burke–Ernzerhof form of the exchange correlation functional was used. A Hubbard U = 2.0 eV was applied to Cr d states, and spin–orbit was included. A plane‐wave cutoff energy of 300 eV was used. A 6 × 6 × 4 Monkhorst–Pack k‐point mesh and the tetrahedron integration method were used. The atomic positions were optimized by the conjugate gradient method to have all forces less than 10^−3^ eV Å^−1^. The in‐plane lattice constant of Cr_2_Te_3_ is 3.8 Å, while out of plane constant is 12.1 Å. Berry curvature analysis and AHE conductivity were calculated using Wannier90. For Wannier interpolation, a k‐mesh of 200 × 200 × 100 and an adaptive mesh of 6 × 6 × 4 were used. The Wannier interpolated band structure accurately recovered the ab‐initio calculated dispersions. The spread for the Wannierization process is converged under 10^−10^ eV Å^2^.

### Heisenberg Model

The Heisenberg model is applied to the frustrated spin lattice to show the possibility of canting and obtain the energy profile in Figure 6e. To simplify the consideration, the study only considers exchange interactions that contribute to spin frustration. The model Hamilton is written as

(1) $$H=-\sum_{i>j}J_{ij}{\vec{S}}_{i}\cdot \;{\vec{S}}_{j}=-2J_{12}\;cos\;\left(\theta \right)\;-6J_{13}cos\;\left(\theta -\tilde{\theta }\right)-6J_{23}cos\;\left(\tilde{\theta }\right)$$

where *J*_12_, *J*_13_ and *J*_23_ are exchange parameters between Cr_I_ and Cr_II_, Cr_I_ and Cr_III_, as well as Cr_II_ and Cr_III_, respectively. θ and $\tilde{\theta }$ are canting angles for Cr_I_ and Cr_III_, respectively, while Cr_II_ is not canted.

## Conflict of Interest

The authors declare no conflict of interest.

## Author Contributions

K.H., M.B., and S.D.S. contributed equally to this work. Y.H. and H.Z. conceived the project. G.M., Y.H., and H.Z. supervised the project. H.R. and R.A. prepared Cr_2_Te_3_ samples. K.H. fabricated Hall devices. M.B. and K.H. performed Hall effect and magneto‐resistivity measurements and did the data analysis. S.S. and P.M. performed MFM measurements. K.J. performed HAADF‐STEM characterizations. A.M., E.K., and S.A.C. performed the MCD measurements. C.Y. and H.X. performed minor AHE loop measurements. J.B., R.S. S.P., J.Y., and J.Z. performed the first‐principal calculations. M.B. and C.H. performed magnetization characterizations. H.H. performed XRD measurement. M.B. performed AFM measurements. K.H., M.B., Y.H., R.S., and H.Z. wrote the manuscript. All authors discussed the results and commented on the manuscript.

## Supporting information



Supporting Information

## Data Availability

The data that support the findings of this study are available from the corresponding author upon reasonable request.
