# Supplementary material for: Unconventional Anomalous Hall Effect Driven by Self‐Intercalation in Covalent 2D Magnet Cr2Te3
Source: Adv Sci (Weinh). 2024 Nov 25;12(2):2407625. doi: 10.1002/advs.202407625 (PMC11727383; doi:10.1002/advs.202407625)
Supplement: Supplementary file 1 — Supporting Information [file ADVS-12-2407625-s001.pdf]

ADVANCED SCIENCE
Open Access

# Supporting Information

for *Adv. Sci.*, DOI 10.1002/advs.202407625

Unconventional Anomalous Hall Effect Driven by Self-Intercalation in Covalent 2D Magnet $Cr_2Te_3$

*Keke He, Mengying Bian, Samuel D. Seddon, Koushik Jagadish, Andrea Mucchietto, He Ren, Erik Kirstein, Reza Asadi, Jaeil Bai, Chao Yao, Sheng Pan, Jie-Xiang Yu, Peter Milde, Chang Huai, Haolei Hui, Jiadong Zang, Renat Sabirianov, Xuemei M. Cheng, Guoxing Miao*, Hui Xing, Yu-Tsun Shao, Scott A. Crooker, Lukas Eng, Yanglong Hou*, Jonathan P. Bird and Hao Zeng**

# Unconventional Anomalous Hall Effect Driven by Self-Intercalation in Covalent 2D Magnet $Cr_2Te_3$

Keke He[1,2]†, Mengying Bian[1,3,13]†, Samuel D. Seddon[4]†, Koushik Jagadish[5], Andrea Mucchietto[6], He Ren[7], Erik Kirstein[6], Reza Asadi[7], Jaeil Bai[8], Chao Yao[9], Sheng Pan[10], Jie-Xiang Yu[10], Peter Milde[4], Chang Huai[1], Haolei Hui[1], Jiadong Zang[11], Renat Sabirianov[8], Xuemei M. Cheng[12], Guoxing Miao[7]*, Hui Xing[9], Yu-Tsun Shao[5], Scott A. Crooker[6], Lukas Eng[4], Yanglong Hou[3,14]*, Jonathan P. Bird[2], and Hao Zeng[1]*

[1]*Department of Physics, University at Buffalo, the State University of New York, Buffalo, NY, 14226, USA*

[2]*Department of Electrical Engineering, University at Buffalo, the State University of New York, Buffalo, NY, 14226, USA*

[3]*School of Materials Science and Engineering, Peking University, Beijing, 100871, China*

[4]*Institute of Applied Physics, Technical University of Dresden, Dresden, 01187, Germany*

[5]*Mork Family Department of Chemical Engineering and Materials Science, University of Southern California, Los Angeles, CA, 90089, USA*

[6]*National High Magnetic Field Laboratory; Los Alamos National Lab; Los Alamos, New Mexico 87545, USA*

[7]*Department of Electrical and Computer Engineering Institute for Quantum Computing, University of Waterloo, Ontario, Canada*

[8]*Department of Physics, University of Nebraska-Omaha, Omaha, NE, 68182, USA*

[9]*Key Laboratory of Artificial Structures and Quantum Control, and Shanghai Center for Complex Physics, School of Physics and Astronomy, Shanghai Jiao Tong University, Shanghai, 200240, China*

[10]*School of Physical science and technology, Soochow University, Suzhou, 215006, China*

[11]*Department of Physics and Astronomy, University of New Hampshire, Durham, NH, 03824, USA*

[12]*Physics Department, Bryn Mawr College, Bryn Mawr, PA, 19010, USA*

[13]*College of Materials Science and Engineering, Beijing University of Technology, Beijing, 100124, China*

[14]*School of Materials, Sun Yat-San University, Shenzhen, 518107, China*

*e-mail: haozeng@buffalo.edu; hou@pku.edu.cn; g2miao@uwaterloo.ca

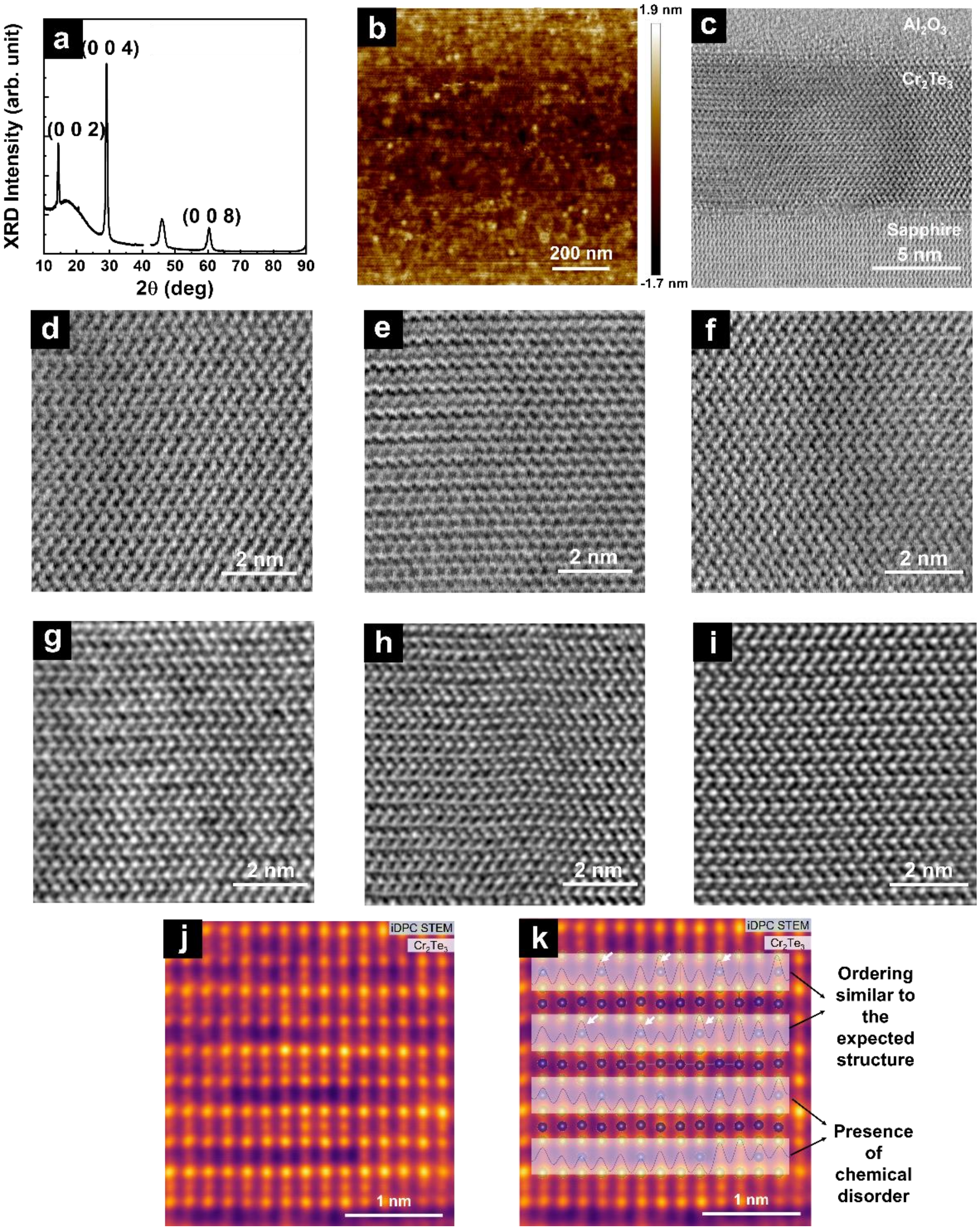


**Figure S1.** Structural characterizations of $Cr_2Te_3/Al_2O_3$ film. a) The XRD pattern of an 8-unit cell (UC) thick MBE $Cr_2Te_3$ film, which matches with the standard XRD pattern for hexagonal structured $Cr_2Te_3$ (PDF#29-0458). An (001) orientation is observed, which is indicative of a single crystalline structure of the as-grown $Cr_2Te_3$ film. b), the AFM image showing the surface morphology of an 8UC MBE $Cr_2Te_3/Al_2O_3$ film, the roughness of which is 0.21 nm. c) A cross-sectional HAADF-STEM image along the [210] zone axis of the $Cr_2Te_3$ film on sapphire substrate with an $Al_2O_3$ capping layer. d)-f) Cross-sectional atomic-resolution HAADF-STEM images and g)-i) the iDPC images along [210] zone axis of $Cr_2Te_3$ from different regions marked by red, green, and blue boxes in Figure 1c. j) An original cross-sectional HAADF-STEM image of the $Cr_2Te_3$ thin film taken along the [100] axis with iDPC technique and k) corresponding line scans in the partially occupied layers.

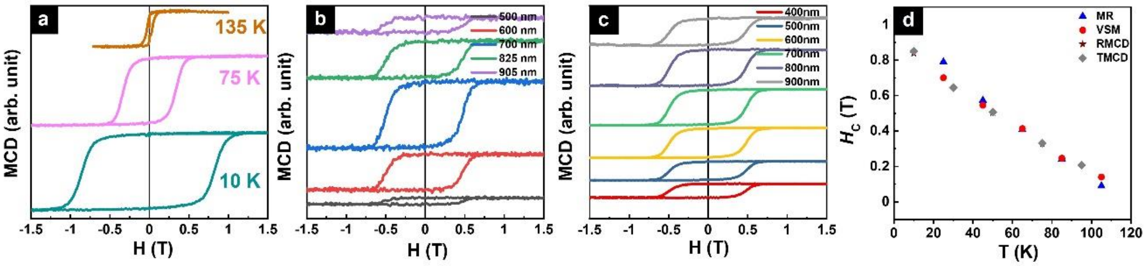


**Figure S2.** MCD measurements of the unannealed 8-unit cell thick $Cr_2Te_3$ thin film. a) Out-of-plane reflective MCD (RMCD) hysteresis loops measured at different temperatures, which are consistent with MCD measured in the transmission mode shown in Figure 1f) (From bottom to top, measurement temperatures are 10 K, 75 K, and 135 K, respectively.) b) Out-of-plane MCD (transmission) and c) RMCD hysteresis loops measured using probe light at different wavelengths. d) $H_C$ as a function of temperature extracted from magnetic (circle), TMCD (square), RMCD (star), and MR (triangle) hysteresis measurements.

## S1 Decomposition method of the two AHE channels

As shown in Figures 4 and 5 in the main text, after subtracting the linear OHE part from the high-field data, the field dependent Hall resistivity observed at different temperatures are fitted using $\rho_{AHE1}$ and $\rho_{AHE2}$ with opposite polarities. For this purpose, the signals were fitted to:

$$\rho_{AHE}\,(H) = \frac{\rho_{AHE1}}{1 + e^{(H \pm H_{C1})/H_{01}}} + \frac{\rho_{AHE2}}{1 + e^{(H \pm H_{C2})/H_{02}}}$$

Here, $\rho_{AHE1}$ ($\rho_{AHE2}$) is the first (second) anomalous Hall resistivity contribution with $H_{C1}$ ($H_{C2}$) being the coercivity and $H_{01}$ ($H_{02}$) defining the squareness of the hysteresis loops, respectively. It can be seen that the fits reproduce the details of the measured data very well.

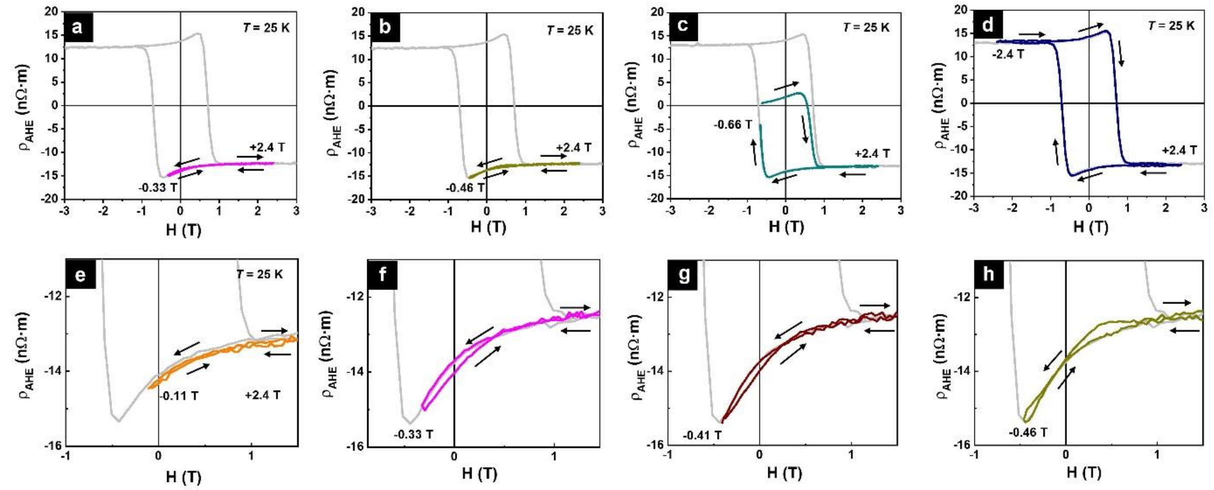


**Figure S3.** Minor AHE hysteresis loops for the unannealed 8-unit cell thick $Cr_2Te_3$ thin film. Minor loops of AHE resistivity measured at 25 K for different stopping fields of a) -0.33 T (magenta curve), b) -0.46 T (dark yellow curve), c) -0.66 T (dark cyan curve), and d) -2.4 T (navy curve). The magnified minor loops of Figure 4a (e), Figure S3a (f), Figure 4b (g) and Figure S3b (h), respectively.

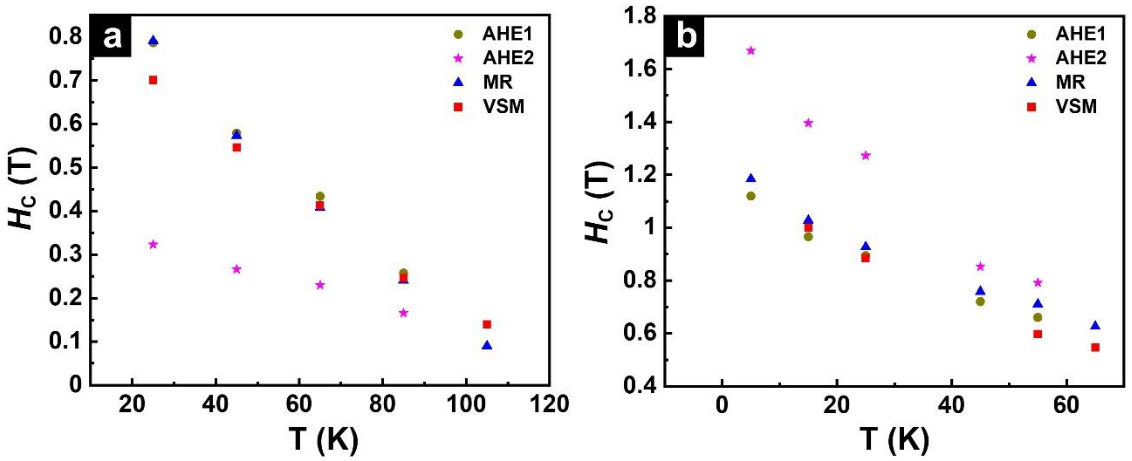


**Figure S4.** Coercivity as a function of temperature. $H_C$ as a function of temperature extracted from AHE1 (circle), AHE2 (star), magnetization (square) and MR (triangle) hysteresis measurements for a) the unannealed sample and b) the annealed sample. For both unannealed and annealed samples, $H_C$ values from AHE1, magnetization and MR hysteresis match with each other, while they do not match with $H_C$ extracted from AHE2 hysteresis.

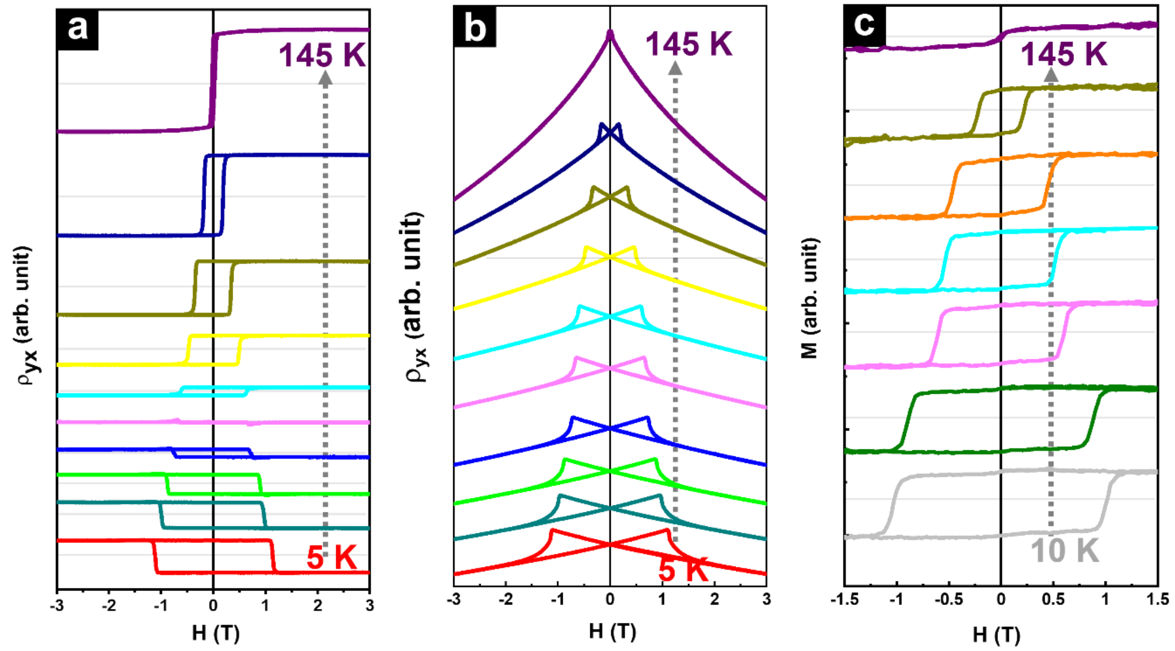


**Figure S5.** AHE resistivity, MR and magnetization measurements of the annealed 8-unit cell thick $Cr_2Te_3$ thin film. a) Shown from bottom to top are the magnetic field dependent AHE resistivity measured at different temperatures of 5 K, 15 K, 25 K, 45 K, 55 K, 65 K, 85 K, 105 K, 125 K and 145 K, respectively. b) Shown from bottom to top are the magnetic field dependent longitudinal resistivity measured at different temperatures of 5 K, 15 K, 25 K, 45 K, 55 K, 65 K, 85 K, 105 K, 125 K, and 145 K, respectively. c) Out-of-plane magnetic hysteresis loops measured at different temperatures (From bottom to top, measurement temperatures are 10 K, 20 K, 55 K, 65 K, 75 K, 105 K, and 145 K, respectively).

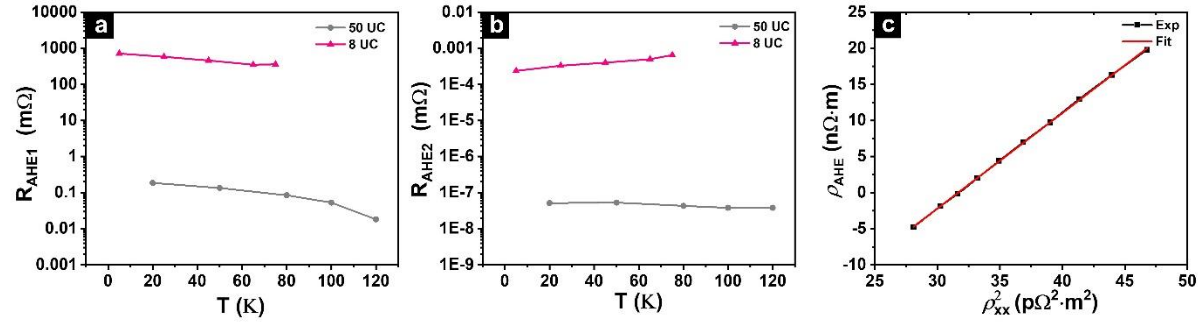


**Figure S6.** Temperature dependence of a) AHE1 resistance ($R_{AHE1}$) and b) AHE2 resistance ($R_{AHE2}$) of 8- and 50-unit cell thick $Cr_2Te_3$ films. $R_{AHE1}$ and $R_{AHE2}$ are of the same order of magnitude for the same film thickness. Both $R_{AHE1}$ and $R_{AHE2}$ depend strongly on thickness. These suggest that both AHE1 and AHE2 originate from the bulk film instead of the interface. c) The AHE resistivity ($\rho_{AHE}$) as a function of longitudinal resistivity ($\rho_{xx}$) measured at zero field for the annealed $Cr_2Te_3$ film, in the temperature range of 25 to 125 K. $\rho_{AHE}$ scales linearly with $\rho_{xx}^2$, suggesting that the AHE in our $Cr_2Te_3$ film is dominated by intrinsic Berry curvature contribution.

## S2 Estimation of the local magnetic moment of chromium atoms

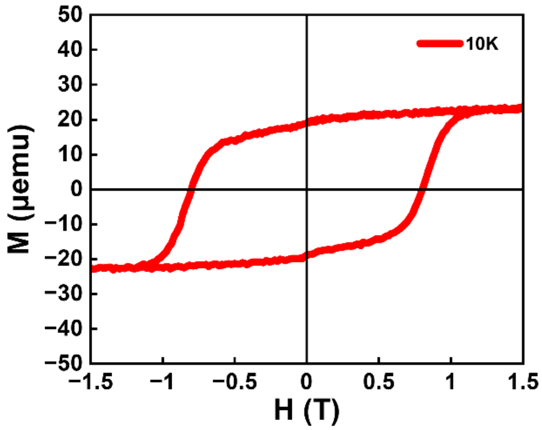


**Figure S7.** Out-of-plane magnetic hysteresis loop measured at 10 K.

For the 8-unit cell (thickness ~ 10 nm) $Cr_2Te_3$ thin film:

- Total saturation magnetic moment at 10 K: $2.29 \times 10^{-5}$ emu
- Film volume: $(8.9 \pm 0.6) \times 10^{-8}$ $cm^3$
- Unit cell volume of $Cr_2Te_3$: $5.58 \times 10^{-22}$ $cm^3$

The estimated value of the magnetic moment is $15.5 \pm 1.0$ $\mu_B$ /unit cell (8 Cr + 12 Te) or ~ $1.94 \pm 0.12$ $\mu_B$/Cr.

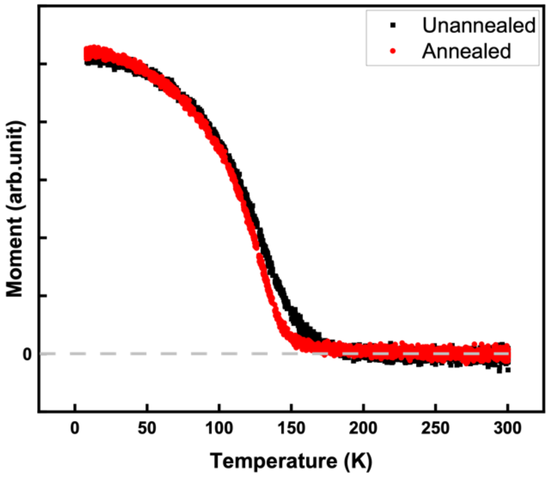


**Figure S8.** Magnetization as a function of temperature for the unannealed (black) and annealed (red) sample.

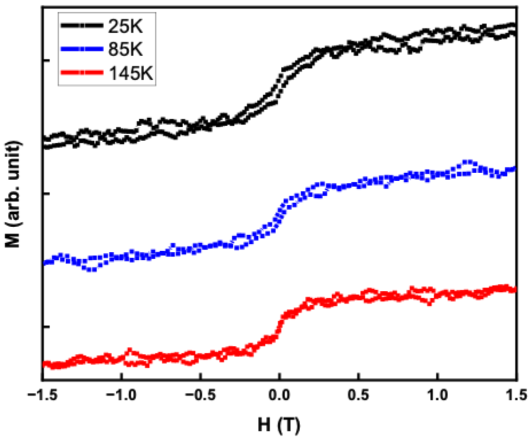


**Figure S9.** In-plane magnetic hysteresis loops of the sample measured at different temperatures.

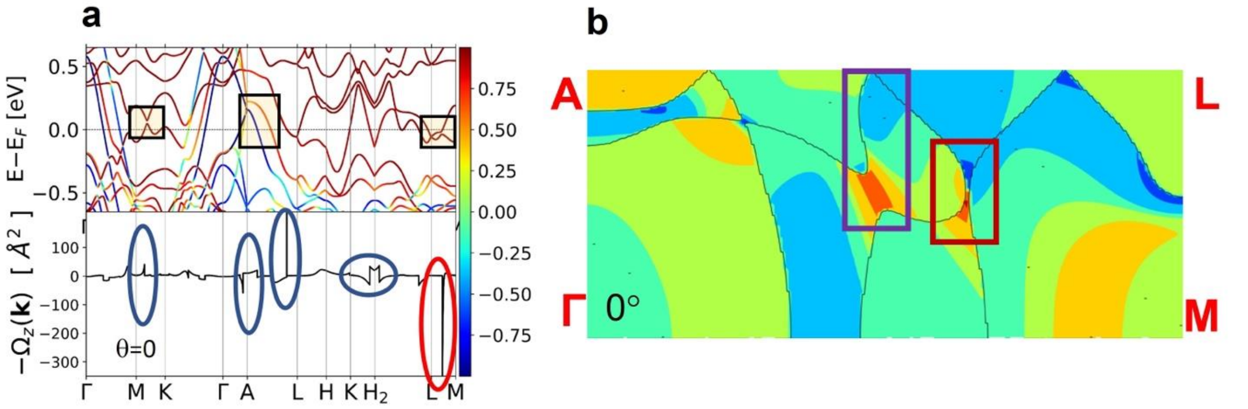


**Figure S10.** Calculated band structure and Berry curvature of $Cr_2Te_3$ in the ferromagnetic configuration. a) Calculated band structure of $Cr_2Te_3$ with $Cr_I$ moment canting angle $\theta = 0$ and corresponding Berry curvature along high-symmetry directions of the Brillouin zone. b) The corresponding surface contour plot of the Berry curvature in the Γ-M-L-A plane.
